# Evaluating the online platform of a blended-learning pharmacist continuing education degree program

**DOI:** 10.3402/meo.v21.31832

**Published:** 2016-06-08

**Authors:** Kerry Wilbur

**Affiliations:** College of Pharmacy, Qatar University, Doha, Qatar

**Keywords:** blended learning, online, courses, peer review, pharmacy education

## Abstract

**Background:**

Distance-based continuing education opportunities are increasingly embraced by health professionals worldwide.

**Methods:**

To evaluate the online component of a blended-learning degree program for pharmacists, we conducted a structured self-assessment and peer review using an instrument systematically devised according to Moore's principles of transactional distance. The web-based platform for 14 courses was reviewed by both local and external faculty, followed by shared reflection of individual and aggregate results.

**Results:**

Findings indicated a number of course elements for modification to enhance the structure, dialog, and autonomy of the student learning experience.

**Conclusion:**

Our process was an important exercise in quality assurance and is worthwhile for other health disciplines developing and delivering distance-based content to pursue.

Maintenance of knowledge and skills is a fundamental responsibility of any health professional involved in the delivery of patient care. Despite concerns regarding its overall utility in changing behavior or health outcomes, participation in continuing education activities (either self-initiated or as a requirement of licensing bodies) remains the principle means for health practitioners to engage in professional development and includes individual, small or large group participation in conferences, lectures, workshops, and rounds (1, 2). The delivery of training through online mediums offers place-bound professionals the opportunity to interact with other adult learners and access expertise from all around the world (3–5). Such educational models are proliferating throughout the world, and universities of prestige are offering online alternatives to overseas and local students alike (6, 7).

A number of theoretical and pedagogical frameworks exist to inform programs and faculty embarking on the development and delivery of distance-based courses, many of which have been proposed even before the Internet became the medium for such instruction. One of the earliest frameworks involves the work of Moore who in the 1970s first described a theory of transactional distance, the communication space between student and instructor that must be negotiated to optimize learning, and is underpinned by three major variables: dialog, structure, and autonomy (8, 9). Dialog is the degree and nature of interaction among the program, the learners, and the educators, whereby structure corresponds to elements of course design and delivery through various media. Autonomy represents the student's ability to determine goals and self-direct learning.

Efforts have been made to link continuing education delivered online with professional performance, but until now assessment of the actual web-based platform for such distance-based education in health disciplines has not been well described (10, 11). There is a large body of literature outlining the processes and merits of peer evaluation of the health professional teacher in university settings (12–15). Similarly, means to evaluate the instructional design of the entire courses themselves have been proposed (16, 17). However, evaluation of the design and delivery of online courses in health professional education is lacking, especially within blended-learning environments combining self-directed asynchronous and synchronous web-based activities with live in-person instruction (18). We sought to develop the means to conduct a peer assessment of the blended learning offered in our graduate pharmacy degree program.

## Methods

### Program context

The Doctor of Pharmacy (PharmD) program at the College of Pharmacy (CPH) at Qatar University (QU) is a post-baccalaureate degree supporting the training of students to assume advanced pharmacy practice positions as integrated members of multidisciplinary teams delivering direct patient care. The QU CPH PharmD program offers a part-time study plan for practicing pharmacists in Qatar who have obtained their pharmacy degree elsewhere. Pharmacists working in the country are a heterogeneous group, with them having graduated from pharmacy programs from all over the region but whose curricula are highly technical and product-oriented versus patient-oriented (19). While full-time PharmD students may enter the 8-month-long internship phase directly upon program enrolment, part-time PharmD students first complete a series of bridging courses over 2 to 3 years.

These bridging courses derive content delivered in the baccalaureate program and offer as a blended-learning experience for pharmacist professionals who are unable to discontinue work to regularly attend live classroom-based courses. Lecture-capture is in place across all pharmacy courses employing Echo360^®^ media platform (Echo360^®^, Dulles, VA) to record audio, video, and computer/data camera images (20). Links to these archived undergraduate recorded lectures are uploaded to the PharmD Blackboard^®^ (course management) website to accompany posted handouts. The part-time PharmD students access this collection and other resources, assignments, and assessments that course coordinators have further tailored to account for the practice experiences of these students. They may review content at their convenience and control the quantity they consume at any given time (within the constructs of a guiding schedule set out at the start of the semester by each course coordinator). Synchronous exercises (such as discussion boards) are also incorporated. Finally, the graduate students make monthly on-campus visits for faculty-led group sessions to complement web-based content and conduct certain live assessments.

### Instrument development

A comprehensive review of available literature was conducted to identify the development or use of an instrument to evaluate the quality of a distance-based course. Electronic databases related to healthcare, education, and technology were searched using predetermined key words or phrases. References of any retrieved articles were additionally hand-searched. Abstracts of unpublished studies were identified by scanning proceedings from relevant conferences. Predetermined search terms were also applied to a general Internet search using Google Scholar. Located tools were reviewed by three separate pharmacy educators who then selected the specific evaluation form to serve as our peer-review tool, or if none were deemed appropriate, the primary sources for development of our own instrument. Any items chosen for extraction to contribute to a compilation were determined by consensus.

### Course review

Using the developed instrument, each course coordinator conducted: 1) a self-assessment of their blended-learning course(s) and 2) a blinded evaluation of at least one blended-learning course of a peer. These internal self-review and peer-review processes were complemented by an external review by faculty with expertise in the delivery of distance-based courses from the University of Bath. Inter-rater reliability was calculated using Krippendorff alpha using the following benchmarks for observed coefficients: *K*<0 ‘poor’ agreement, 0 to 0.2 ‘slight’, 0.21 to 0.40 ‘fair’, 0.41 to 0.60 ‘moderate’, 0.61 to 0.80 ‘substantial’, and 0.81 to 1 ‘near perfect’ (21). Paired comparisons among peers (QU and University of Bath) and faculty (QU self and peer) were evaluated using Cohen's alpha coefficient.

## Results

Numerous existing instruments were identified by our search strategy but none described rigorous methods of validation. The content of four main instruments was used to model a 73-item tool (Supplementary file). Featured categories for online course assessment included: 1) instructional design; 2) communication, interaction, and collaboration; 3) student evaluation and assessment; 4) learner support and resources; 5) web design; and 6) course evaluation. Resultant item dimensions blueprinted to all constructs of transactional distance (Table 1). Assigned judgments of each described item were made according to an interval scale with four categories ranging from ‘exemplary model’ share with others and ‘meets criteria’ requiring no revision to ‘partially meets criteria’ and ‘does not meet criteria’ requiring revision. A fifth category to indicate ‘not applicable’ or ‘not enough information’ was available to offer a score.

**Table 1:** Online course peer-review instrument items linked to principles of distance-based learning

Principles	Developed instrument	

Distance-based learning	Category	Subcategory
Dialog	Instructional design	Course information Structure Instructional strategies
	Communication, interaction, and collaboration	All subcategories
	Student evaluation and assessment	Goals and objectives Strategies Grades Feedback Management
	Course evaluation	
Structure	Instructional design Communication,	All subcategories
	Interaction and collaboration Student evaluation and assessment	Organization and management
	Learner support and resources Web design Course evaluation	All subcategories All subcategories All subcategories
Autonomy	Instructional design	Course information Structure
	Learner support and resources	Institutional/program support and resources Academic support and resources

From Moore (8), Chickering & Gamson (22).

Seven internal faculty members and two external faculty peers reviewed 14 courses. No singular course was broadly deficient and common strengths and weaknesses arose across all courses.

### Instructional design

Course information (its description and content overview, instructors, materials to be used over the semester) was well outlined. However, described technical requirements and competencies necessary to complete the course were largely lacking. Most courses did not post a singular calendar of due dates or on-campus sessions, but these instead were embedded elsewhere throughout the course site. Course objectives were well articulated but alignment with specific assignments was not always found. Specifically, purposes for asynchronous (web-based) and on-campus (live) learning activities and the relationship between the two were not consistently documented.

### Communication, interaction, and collaboration

Asynchronous group work was minimal, although some courses promoted student–student communication through discussion board activities. Formal student–student and student–faculty interactions appeared mostly reserved for on-campus sessions.

### Student evaluation and assessment

Varied instructional delivery methods (e.g., lecture, demonstration, discovery, and group work), including audio and visual multimedia, were employed, and all courses were organized so as to permit students to demonstrate their knowledge by various means (quizzes, discussions, and projects). Assessment deadlines were generally evenly distributed across the semester and rubrics for grading posted when available in most courses. However, neither information regarding the consequences of late or incomplete submissions nor standardized processes for feedback was (the mechanism or the timeline) apparent.

### Learner support and resources

While the inventory of academic supports and resources, including the library, tutoring and, student counseling services, was easily located, links to institutional and program policies and procedures were not always provided. Furthermore, students were not clear about the steps or measures to take when the need for technical support arose.

### Web design, course evaluation

The layout and navigation of all courses were consistent with good-quality handouts and lectures (audio–video) at appropriate file sizes for viewing of downloads. The processes for course and instructor evaluations were clearly in place according to university-wide mechanisms.

The overall level of agreement between reviewers, as well as between intracollegiate peers and intercollegiate colleagues, was fair (Table 2). Following the evaluation period, coordinators received the blinded peer reviews of their courses and, paired with their own self-assessment, were asked to reflect on the findings and propose action plans for change. The participating faculty then convened as a group to share and deliberate ideas to implement in the program. A series of revisions were made across all courses to resolve specific identified deficiencies (Table 3).

**Table 2:** Level of agreement among self-review and peer review

Course	K alpha (all raters)	Cohen alpha (peer-paired raters)	Cohen alpha (QU-paired raters)
Critical appraisal I	0.42	0.28	0.35
Professional skills I	0.34	0.40	0.31
Pathophysiology	0.30	0.03	0.42
Pharmacotherapy I	0.49	0.42	0.53
Interpretation of lab data I	0.24	0.15	0.40
Physical assessment I	0.38	0.38	0.16
Critical appraisal II	0.30	0.97	0.25
Professional skills II	0.40	0.22	0.25
Pharmacotherapy II	0.30	0.40	0.19
Interpretation of lab data II	0.39	0.49	0.30
Physical assessment II	0.40	0.45	0.40
Critical appraisal III	0.40	0.40	0.39
Professional skills III	0.31	0.37	0.12
Pharmacotherapy III	0.30	0.33	0.25

Cohen alpha coefficient was used for paired raters and Krippendorff alpha coefficient for more than two raters. Judgments were compared according to rated items that ‘do not meet criteria’.

**Table 3:** Recommendations to improve the blended-learning program

**Dialog**
Embed an instructor welcome video in the course website
Devise an online icebreaker at the start of each course
Program-wide and course-wide messages to go to all student email accounts
Capitalize on teaching assistant time and expertise to support course maintenance
Communicate clear grading policies and penalties for late submission
Reinforce student codes of conduct and academic integrity
**Structure**
Post required technical competencies and review at first in-person opportunity
Routine checking of integrity of posted content and media links
Increase use of rubrics in assessment
Share examples and model answers for most assignments
**Autonomy**
Create a program-wide calendar of all course activities and deadlines
Communicate on-campus session expectations in a timely and prospective fashion

## Discussion

Our study is the first known evaluation of the online platform of blended-learning courses in pharmacist continuing education degree programs. Following a comprehensive search at the time of our work, no validated rubric was identified; however, many examples of assessment tools existed. The instrument we subsequently developed encompassed the features widely considered necessary to assess transactional distance as a measure of program strengths and weaknesses. Use of this tool through course coordinator self-assessment and peer review by local and overseas faculty identified a number of aspects meriting attention.

Observed course deficiencies may be broadly categorized as failures in communication. First, lack of described baseline technical requirements, evidence of broken or missing content links, and unclear direction for students to alert and seek resolution of technical difficulties they may encounter can compromise student learning. Frustration with the inability to timely access materials and the resultant negative impact on course engagement, retention, and satisfaction is well-documented among distance-based learners (23, 24). Adult learners are typically more familiar with the passive, classroom formats of their prior learning environments and potentially possess anxieties related to technologies or web-based media (21, 25). Adequate institutional information technology infrastructure must be in place for both student and faculty support. Second, potential ambiguity in the instructions for asynchronous activity was detected. Clear explanations are particularly critical for place-bound learners who cannot routinely clarify directions by the usual in-person encounters that occur with greater ease in live programming. Faculty workload associated with student demands (like these academic inquiries or otherwise) is frequently underestimated and further underscores the importance of well-described tasks and assignments with worked-out examples when possible (26). Having one calendar integrating all course and program deadlines can facilitate student organization and self-regulation. Third, overt linkage between distance-based content and on-campus activities was not always present. Students in blended-learning programs such as ours are working pharmacists and so all elements of course design should strive to incorporate meaningful and realistic problems in order to scaffold prior learning. In particular, programming live, in-person sessions to reinforce asynchronous content with further examples and practice are also important opportunities to prompt students to link and contrast course materials with experiences and beliefs (25, 27).

The program and faculty challenges faced when adoption of distance-based teaching and learning occur within a curriculum have been well described (26, 28). Instructors may be content experts capable of outlining a traditional lesson plan but are not necessarily inherently equipped with the skills required to deliver online courses in an effective fashion. New competencies in online pedagogy and technology must be complemented by positive attitudes toward online teaching and learning. Interestingly, our best-reviewed course was heavily influenced by the coordinator's own experience as a distance-based learner voluntarily enrolled in a massive open online course (MOOC). Institutional support for faculty professional development, as well as recognition of the time required for course preparation and maintenance is essential for quality blended-learning programming (26).

Peer review is a readily accepted means to explore and subsequently improve the quality of teaching through scholarly judgments. Our study method was one of collaborative peer review whereby formative feedback is generated through respectful inquiry and dialog to promote reciprocal learning and professional development. It serves as a valuable exercise for collegial exchange and breaking of ‘pedagogical solitude’ (29). We sought to replicate known conditions for effective online course content review, including the use of a resource tool within a climate of trust; all coordinators serving as peer reviewers also subjected their own course for assessment. Responsibility for course feedback was shared across teams of internal faculty and external members. Perspectives of non-content experts offered the additional advantage of unfamiliarity with navigating the course platform, not unlike a new student would face (29). Other research attempting to compare judgments among peer-review teams could not be identified and disappointingly, the level of agreement in our study was not strong. The devised instrument was lengthy and orientation to its application disparate (e.g., the peer-review team did not work through an example together). Still, this is a useful field of inquiry in peer-review methodologies for others to pursue given that collective opinions of colleagues from diverse contexts may outweigh the value of impressions by a single expert.

Our process is an important exercise for quality assurance and the evaluation tool a vehicle for training new instructors when they assume blended-learning course coordinator roles. Like any course, complete assessment of our blended-learning programming must next incorporate the perspectives of its enrolled students (30).

## Conclusion

Given the proliferation of distance-based courses and degrees, it may no longer be considered a ‘non-traditional’ form of learning. Structured self-assessment and internal and external peer reviews with reflection are valuable exercises to identify means for faculty to augment development and delivery of blended-learning courses.

## Supplementary Material

Evaluating the online platform of a blended-learning pharmacist continuing education degree program
