# Supplementary material for: Evaluating the online platform of a blended-learning pharmacist continuing education degree program
Source: Med Educ Online. 2016 Jun 8;21:10.3402/meo.v21.31832. doi: 10.3402/meo.v21.31832 (PMC4901507; doi:10.3402/meo.v21.31832)
Supplement: Evaluating the online platform of a blended-learning pharmacist continuing education degree program [file MEO-21-31832-s001.pdf]

Course: Course Coordinator/s:
AY Delivered: Self-Assessment:
Students Completed: Peer Assessment:

**SECTION 1**	**Instructional Design**<br>Instructional Design refers to the analysis of learning needs and the systematic approach of developing an online course in a manner that facilitates the transfer of knowledge and skills to the learner through the use of a variety of instructional methods, which relate to multiple learning styles, strategies, and preferences.			
**A. Course Information**				
Description	*A course description is provided.*			
Not enough information or not applicable	Does not meet criteria & needs revision	Partially meets criteria & needs revision	Meets criteria No revision required	Exemplary model Share with others
Instructor Information	*Instructor information is available to student with contact, biographical, and availability information.*			
Not enough information or not applicable	Does not meet criteria & needs revision	Partially meets criteria & needs revision	Meets criteria No revision required	Exemplary model Share with others
Instructional Materials	*Students are provided with a list of supplies such as textbooks and other instructional materials that will be used in the course*			
Not enough information or not applicable	Does not meet criteria & needs revision	Partially meets criteria & needs revision	Meets criteria No revision required	Exemplary model Share with others
Credit Hours	*Course provides information regarding number of credit hours earned upon the successful completion.*			
Not enough information or not applicable	Does not meet criteria & needs revision	Partially meets criteria & needs revision	Meets criteria No revision required	Exemplary model Share with others
Content Overview	*A clear, concise list of activities that will be completed throughout the course with ties to specific content is provided.*			
Not enough information or not applicable	Does not meet criteria & needs revision	Partially meets criteria & needs revision	Meets criteria No revision required	Exemplary model Share with others

Grading Policy	*A distribution of grading scale and weights for all course activities/assessments is (prospectively) provided.*			
Not enough information or not applicable	Does not meet criteria & needs revision	Partially meets criteria & needs revision	Meets criteria No revision required	Exemplary model Share with others
Calendar	*A calendar of due dates and other events for the semester is provided within the course.*			
Not enough information or not applicable	Does not meet criteria & needs revision	Partially meets criteria & needs revision	Meets criteria No revision required	Exemplary model Share with others
Technical Competencies	*A list of technical competencies necessary for course completion is provided.*			
Not enough information or not applicable	Does not meet criteria & needs revision	Partially meets criteria & needs revision	Meets criteria No revision required	Exemplary model Share with others
Technical Requirements	*A list of technical requirements such as connection speed, hardware, and software is provided.*			
Not enough information or not applicable	Does not meet criteria & needs revision	Partially meets criteria & needs revision	Meets criteria No revision required	Exemplary model Share with others
**B. Learning Outcomes**				
Course Objectives	*The course learning outcomes are measurable and clearly state what the participants will know or be able to do at the end of the course; they are linked to institutional (College-level) outcomes.*			
Not enough information or not applicable	Does not meet criteria & needs revision	Partially meets criteria & needs revision	Meets criteria No revision required	Exemplary model Share with others
Course Alignment	*The course content and assessment are aligned with course learning outcomes and the relationship among them is clearly indicated.*			
Not enough information or not applicable	Does not meet criteria & needs revision	Partially meets criteria & needs revision	Meets criteria No revision required	Exemplary model Share with others
**C. Structure**				
Sequence	*The course content is sequenced and structured in a manner that enables learners to achieve the stated goals.*			
Not enough information or not applicable	Does not meet criteria & needs revision	Partially meets criteria & needs revision	Meets criteria No revision required	Exemplary model Share with others

Chunking	*The course material is appropriately "chunked" or grouped to help students learn the content with activities frequently interspersed that involve both content recall and application.*			
Not enough information<br>or not applicable	Does not meet criteria<br>& needs revision	Partially meets criteria<br>& needs revision	Meets criteria<br>No revision required	Exemplary model<br>Share with others
Purpose - Online	*The purpose of each asynchronous learning activity is clearly presented among the posted course materials.*			
Not enough information<br>or not applicable	Does not meet criteria<br>& needs revision	Partially meets criteria<br>& needs revision	Meets criteria<br>No revision required	Exemplary model<br>Share with others
Purpose – Face-to-Face	*The purpose of each on-campus learning activity is clearly presented among the posted course materials.*			
**Not enough information<br>or not applicable**	Does not meet criteria<br>& needs revision	Partially meets criteria<br>& needs revision	Meets criteria<br>No revision required	Exemplary model<br>Share with others
On-campus reinforcement	*On-campus activity reinforces course content.*			
Not enough information<br>or not applicable	Does not meet criteria<br>& needs revision	Partially meets criteria<br>& needs revision	Meets criteria<br>No revision required	Exemplary model<br>Share with others
On-campus follow-up	*On-campus activities are followed-up with reinforcing or "next level" asynchronous activity.*			
Not enough information<br>or not applicable	Does not meet criteria<br>& needs revision	Partially meets criteria<br>& needs revision	Meets criteria<br>No revision required	Exemplary model<br>Share with others
**D. Instructional Strategies**				
Multimodal Instruction	*A variety of instructional delivery methods, accommodating multiple learning styles, are available throughout the course.*			
Not enough information<br>or not applicable	Does not meet criteria<br>& needs revision	Partially meets criteria<br>& needs revision	Meets criteria<br>No revision required	Exemplary model<br>Share with others
Knowledge Demonstration	*A variety of ways (asynchronous + face-to-face) for learners to demonstrate knowledge is provided. Examples may include: examples; submitted case studies; simulations; reading and writing activities; discussions.*			
Not enough information<br>or not applicable	Does not meet criteria<br>& needs revision	Partially meets criteria<br>& needs revision	Meets criteria<br>No revision required	Exemplary model<br>Share with others

Ice Breaker	*At the beginning of the course, students and the instructor are provided with an opportunity to introduce themselves to each other as a way of encouraging synergy within the course.*			
Not enough information or not applicable	Does not meet criteria & needs revision	Partially meets criteria & needs revision	Meets criteria No revision required	Exemplary model Share with others
**E. Academic Integrity**				
Code of Conduct	*Academic integrity and netiquette (internet etiquette) expectations regarding course activities, discussions, email communications and plagiarism are clearly stated.*			
Not enough information or not applicable	Does not meet criteria & needs revision	Partially meets criteria & needs revision	Meets criteria No revision required	Exemplary model Share with others
**F. Use of Multimedia**				
Audio	*Audio files have a specific purpose that does not distract from course goals and objectives.*			
Not enough information or not applicable	Does not meet criteria & needs revision	Partially meets criteria & needs revision	Meets criteria No revision required	Exemplary model Share with others
Video	*Video files have a specific purpose that does not distract from course goals and objectives.*			
Not enough information or not applicable	Does not meet criteria & needs revision	Partially meets criteria & needs revision	Meets criteria No revision required	Exemplary model Share with others
Additional Comments related to Instructional Design				

**SECTION 2**	**Communication, Interaction, & Collaboration**<br>Communication, Interaction, & Collaboration addresses how the course design, assignments, and technology effectively encourage exchanges among the instructor, students, and content.			
**A. Activities and Opportunities**				
Student-Student	*Course design of learning activities and other opportunities for appropriate student-student communication and interaction to foster attainment of learning outcomes and application of the material: For example, collaborative learning opportunities are available through email, discussion boards, group projects, peer assessments and/or on-campus activities. These learning activities also provide a plan for some element of instructor monitoring of these interactions.*			
Not enough information<br>or not applicable	Does not meet criteria<br>& needs revision	Partially meets criteria<br>& needs revision	Meets criteria<br>No revision required	Exemplary model<br>Share with others
Student-Instructor	*Course design of learning activities and other opportunities are developed to foster student-instructor communication and/or collaboration.*			
Not enough information<br>or not applicable	Does not meet criteria<br>& needs revision	Partially meets criteria<br>& needs revision	Meets criteria<br>No revision required	Exemplary model<br>Share with others
Support and Facilitation	*Instructors respond to students in a timely fashion and feedback may include emails, posts to course site or on-campus discussions.*			
Not enough information<br>or not applicable	Does not meet criteria<br>& needs revision	Partially meets criteria<br>& needs revision	Meets criteria<br>No revision required	Exemplary model<br>Share with others
Student-Content	*Course design and learning activities facilitates student interaction with the content to foster attainment of learning objectives and application of materials/content. Students have access to appropriate resources that enrich course content and are aligned with course objectives.*			
Not enough information<br>or not applicable	Does not meet criteria<br>& needs revision	Partially meets criteria<br>& needs revision	Meets criteria<br>No revision required	Exemplary model<br>Share with others

B. Organization and Management				
Types	*Course clearly offers separate 'forums' for description, content, assignments, assessments, discussion, and questions*			
Not enough information or not applicable	Does not meet criteria & needs revision	Partially meets criteria & needs revision	Meets criteria No revision required	Exemplary model Share with others
Organization	*Discussions are organized in clearly defined forums and/or threads.*			
Not enough information or not applicable	Does not meet criteria & needs revision	Partially meets criteria & needs revision	Meets criteria No revision required	Exemplary model Share with others
Access	*Access is available to individuals or groups based on the discussion's purpose, such as private conversations between student and instructor, group work, and class interactions.*			
Not enough information or not applicable	Does not meet criteria & needs revision	Partially meets criteria & needs revision	Meets criteria No revision required	Exemplary model Share with others
Role	*The instructor's role in discussion activities/forums is clearly defined.*			
Not enough information or not applicable	Does not meet criteria & needs revision	Partially meets criteria & needs revision	Meets criteria No revision required	Exemplary model Share with others
**C. Group Work**				
Task	*A statement of the group's overall task is provided, with clear and concise outcomes that are appropriate, reasonable, and achievable.*			
Not enough information or not applicable	Does not meet criteria & needs revision	Partially meets criteria & needs revision	Meets criteria No revision required	Exemplary model Share with others
Formation	*Rules for forming groups and assigning roles within each are clearly stated.*			
Not enough information or not applicable	Does not meet criteria & needs revision	Partially meets criteria & needs revision	Meets criteria No revision required	Exemplary model Share with others
Management	*Benchmarks and expectations of group participation are clearly stated.*			
Not enough information or not applicable	Does not meet criteria & needs revision	Partially meets criteria & needs revision	Meets criteria No revision required	Exemplary model Share with others

Delivery	*A statement of how, when, and where the final project will be delivered is provided.*			
Not enough information or not applicable	Does not meet criteria & needs revision	Partially meets criteria & needs revision	Meets criteria No revision required	Exemplary model Share with others
Additional comments regarding Communication, Interaction, and Collaboration				
SECTION 3	**Student Evaluation and Assessment** Student Evaluation and Assessment refers to the process the course uses to determine student achievement and quality of work, including assigning of grades.			
**A. Goals and Objectives**				
Aligned	*Student evaluation and assessment strategies are consistent with course goals and objectives and are representative of the scope of the course.*			
Not enough information or not applicable	Does not meet criteria & needs revision	Partially meets criteria & needs revision	Meets criteria No revision required	Exemplary model Share with others
Communicated	*Student evaluation and assessment strategies are stated, including clear expectations and criteria for assignments.*			
Not enough information or not applicable	Does not meet criteria & needs revision	Partially meets criteria & needs revision	Meets criteria No revision required	Exemplary model Share with others

**B. Strategies**				
Method	*The course structure includes appropriate methods and procedures to assess student's attainment of intended learning outcomes (for example quizzes, tests, discussions, projects).*			
Not enough information or not applicable	Does not meet criteria & needs revision	Partially meets criteria & needs revision	Meets criteria No revision required	Exemplary model Share with others
Frequency	*The course structure includes adequate methods and procedures to assess student's attainment of intended learning outcomes; imbedded assessments and other strategies are in place throughout the course to ensure accurate and up-to-date assessment and support of student learning.*			
Not enough information or not applicable	Does not meet criteria & needs revision	Partially meets criteria & needs revision	Meets criteria No revision required	Exemplary model Share with others
**C. Grades**				
Rubric	*Grading rubrics specifying criteria and (partially or fully completed) models of assignments demonstrating characteristics of quality expected are provided.*			
Not enough information or not applicable	Does not meet criteria & needs revision	Partially meets criteria & needs revision	Meets criteria No revision required	Exemplary model Share with others
Grading Scale	*The grade values given to assessment and assignments are explicit and balanced to reflect their importance in assessing attainment of learning outcomes and importance of the content being assessed.*			
Not enough information or not applicable	Does not meet criteria & needs revision	Partially meets criteria & needs revision	Meets criteria No revision required	Exemplary model Share with others
Penalties	*Penalties assessed to grades for late submission, for example, are provided.*			
Not enough information or not applicable	Does not meet criteria & needs revision	Partially meets criteria & needs revision	Meets criteria No revision required	Exemplary model Share with others
Participation	*Student participation in asynchronous and on-campus course activities, if applicable, are defined and a mechanism for measurement is provided.*			
Not enough information or not applicable	Does not meet criteria & needs revision	Partially meets criteria & needs revision	Meets criteria No revision required	Exemplary model Share with others

Extra Credit	*A statement describing whether or not extra credit is available is provided, and any extra credit assignments are described in detail, including where, when, and how any extra credit will be administered and submitted.*			
Not enough information or not applicable	Does not meet criteria & needs revision	Partially meets criteria & needs revision	Meets criteria No revision required	Exemplary model Share with others
**D. Feedback**				
When	*Statements explaining when students can expect feedback following each course activity and assessment are provided.*			
Not enough information or not applicable	Does not meet criteria & needs revision	Partially meets criteria & needs revision	Meets criteria No revision required	Exemplary model Share with others
What/How	*Statements explaining the nature of the feedback students can expect following each course activity and assessment are provided.*			
Not enough information or not applicable	Does not meet criteria & needs revision	Partially meets criteria & needs revision	Meets criteria No revision required	Exemplary model Share with others
**E. Management**				
Time and Deadlines	*The course requirements include an approximate time required for individual activities or assessments such as deadlines for online discussion posts and other assignments or exercises.*			
Not enough information or not applicable	Does not meet criteria & needs revision	Partially meets criteria & needs revision	Meets criteria No revision required	Exemplary model Share with others
Availability	*A date/time when each assessment will be available is provided.*			
Not enough information or not applicable	Does not meet criteria & needs revision	Partially meets criteria & needs revision	Meets criteria No revision required	Exemplary model Share with others
Retake	*A statement indicating whether or not the assessment can be retaken is provided.*			
Not enough information or not applicable	Does not meet criteria & needs revision	Partially meets criteria & needs revision	Meets criteria No revision required	Exemplary model Share with others
Delivery Method	*A description of the assessment delivery method is provided.*			
Not enough information or not applicable	Does not meet criteria & needs revision	Partially meets criteria & needs revision	Meets criteria No revision required	Exemplary model Share with others

Submit	*Instructions for completion and submission are clearly provided.*			
Not enough information or not applicable	Does not meet criteria & needs revision	Partially meets criteria & needs revision	Meets criteria No revision required	Exemplary model Share with others
Additional Comments regarding Student Assessment and Evaluation				
SECTION 4	**Learner Support & Resources**<br>Learner Support & Resources refers to the program, academic, and/or technical resources available to learners.			
**A. Institutional/Program Support and Resources**				
Policies	*Links to institutional (QU) and program (QU CPH) information and/or policies and procedures are provided.*			
Not enough information or not applicable	Does not meet criteria & needs revision	Partially meets criteria & needs revision	Meets criteria No revision required	Exemplary model Share with others
Technical Support	*Tools and instructions for viewing course content (browser settings, Adobe, Java, etc.) and steps to take when technical problems arise are provided.*			
Not enough information or not applicable	Does not meet criteria & needs revision	Partially meets criteria & needs revision	Meets criteria No revision required	Exemplary model Share with others

**B. Academic Support and Resources**				
Orientation	*Opportunities for program and course orientation are provided.*<br>*(Note: This is conducted in person at the first on-campus session each semester).*			
Not enough information<br>or not applicable	Does not meet criteria<br>& needs revision	Partially meets criteria<br>& needs revision	**Meets criteria**<br>**No revision required**	Exemplary model<br>Share with others
Resources	*A list of academic resources with links to the institution's library, tutoring center, counseling services and other resources is provided.*			
Not enough information<br>or not applicable	Does not meet criteria<br>& needs revision	Partially meets criteria<br>& needs revision	Meets criteria<br>No revision required	Exemplary model<br>Share with others
Gradebook	A grade book is available for students to check progress.			
Not enough information<br>or not applicable	Does not meet criteria<br>& needs revision	Partially meets criteria<br>& needs revision	**Meets criteria**<br>**No revision required**	Exemplary model<br>Share with others
Additional Comments related to Learner Support & Resources				

SECTION 5	**Web Design**<br>Web Design refers to the use of Web pages, graphics, multimedia, and accessibility standards in the web pages of a course, which are under the course developer's control.			
**A. Layout/Design**				
Scrolling	*Scrolling is minimized or facilitated with anchors.*			
Not enough information or not applicable	Does not meet criteria & needs revision	Partially meets criteria & needs revision	Meets criteria No revision required	Exemplary model Share with others
Consistency	*Consistent layout design orients users throughout the course site.*			
Not enough information or not applicable	Does not meet criteria & needs revision	Partially meets criteria & needs revision	Meets criteria No revision required	Exemplary model Share with others
Fonts	*Font type, size, and color are readable and consistent throughout the course site.*			
Not enough information or not applicable	Does not meet criteria & needs revision	Partially meets criteria & needs revision	Meets criteria No revision required	Exemplary model Share with others
Pop-Up Windows	*Use of pop-up windows (windows with specific information, no scroll bars, and no menus) is appropriate.*			
Not enough information or not applicable	Does not meet criteria & needs revision	Partially meets criteria & needs revision	Meets criteria No revision required	Exemplary model Share with others
Frames	*Windows open in appropriate frames that do not confuse users. The use of additional frames, other than those within the course management software, is avoided.*			
Not enough information or not applicable	Does not meet criteria & needs revision	Partially meets criteria & needs revision	Meets criteria No revision required	Exemplary model Share with others
**B. Use of Multimedia**				
Technical Requirements	*Audio/visual hardware requirements do not extend beyond the basic sound cards, speakers, and video players unless appropriately needed to meet course goals and objectives.*			
Not enough information or not applicable	Does not meet criteria & needs revision	Partially meets criteria & needs revision	Meets criteria No revision required	Exemplary model Share with others

Audio Standards	*Quality of audio files is clear and length is adequate to meet the goals of activity; audio player required is compatible with multiple operating systems.*			
Not enough information or not applicable	Does not meet criteria & needs revision	Partially meets criteria & needs revision	Meets criteria No revision required	Exemplary model Share with others
Video Standards	*Quality of video files is clear and length is adequate to meet the goals of activity; audio player required is compatible with multiple operating systems.*			
Not enough information or not applicable	Does not meet criteria & needs revision	Partially meets criteria & needs revision	Meets criteria No revision required	Exemplary model Share with others
**C. Handouts**				
Image quality	*Content of posted handouts are clear.*			
Not enough information or not applicable	Does not meet criteria & needs revision	Partially meets criteria & needs revision	Meets criteria No revision required	Exemplary model Share with others
File size	*Content files are optimized for efficient loading.*			
Not enough information or not applicable	Does not meet criteria & needs revision	Partially meets criteria & needs revision	Meets criteria No revision required	Exemplary model Share with others
**D. Navigation**				
Consistency	*Navigation aids are located in the same location; graphics used as links are consistent.*			
Not enough information or not applicable	Does not meet criteria & needs revision	Partially meets criteria & needs revision	Meets criteria No revision required	Exemplary model Share with others
Hyperlink identity	*Navigation cues are present, clearly identifiable, offered in text and graphic formats, and are obvious links based on visual cues, such as color, underlining, and text directives (e.g., start here).*			
Not enough information or not applicable	Does not meet criteria & needs revision	Partially meets criteria & needs revision	Meets criteria No revision required	Exemplary model Share with others
Hyperlink function	*Course has no broken links.*			
Not enough information or not applicable	Does not meet criteria & needs revision	Partially meets criteria & needs revision	Meets criteria No revision required	Exemplary model Share with others
Hyperlink target	*Hyperlinks open in appropriate windows or frames.*			
Not enough information or not applicable	Does not meet criteria & needs revision	Partially meets criteria & needs revision	Meets criteria No revision required	Exemplary model Share with others

Additional Comments related to Web Design				
**SECTION 6**	**Course Evaluation**<br>Course Evaluation refers to the processes and mechanism used to elicit feedback from learners for the purpose of course improvement			
Physical Course	*Opportunities for learner feedback throughout the course on issues surrounding the course's physical structure (e.g. spelling mistakes, navigation, deadlinks, missing documents, etc.) are provided.*			
Not enough information or not applicable	Does not meet criteria & needs revision	Partially meets criteria & needs revision	Meets criteria No revision required	Exemplary model Share with others
Instruction	*Opportunities for learners to offer feedback to instructor on instructional strategies are provided.*			
Not enough information or not applicable	Does not meet criteria & needs revision	Partially meets criteria & needs revision	Meets criteria No revision required	Exemplary model Share with others
Content	*Opportunities for learners to offer feedback on course content are provided.*			
Not enough information or not applicable	Does not meet criteria & needs revision	Partially meets criteria & needs revision	Meets criteria No revision required	Exemplary model Share with others
Additional Comments Related to Course Evaluation				

Compiled and Adapted from:

Quality Online Course Initiative Rubric by Illinois Online Network (licensed under a Creative Commons Attribution-Noncommercial-Share Alike 3.0 United States License.

Checklist for Evaluating Online Courses. Southern Regional Education Board Educational Technologoy Cooperative (November 2006).

Online Course Development Guide and Rubric. Michigan Community College Association Virtual Learning Collaborative.

Online Course Development Guide and Rubric. University of Southern Mississippi Learning Enhancement Center.
